# Focal white matter disruptions along the cingulum tract explain cognitive decline in amnestic mild cognitive impairment (aMCI)

**DOI:** 10.1038/s41598-020-66796-y

**Published:** 2020-06-23

**Authors:** Elveda Gozdas, Hannah Fingerhut, Lindsay C. Chromik, Ruth O’Hara, Allan L. Reiss, S. M. Hadi Hosseini

**Affiliations:** Department of Psychiatry and Behavioral Sciences, Stanford University School of Medicine, Stanford, CA USA

**Keywords:** Neuroscience, Cognitive ageing

## Abstract

White matter abnormalities of the human brain are implicated in typical aging and neurodegenerative diseases. However, our understanding of how fine-grained changes in microstructural properties along white matter tracts are associated with memory and cognitive decline in normal aging and mild cognitive impairment remains elusive. We quantified tract profiles with a newer method that can reliably measure fine-grained changes in white matter properties along the tracts using advanced multi-shell diffusion magnetic resonance imaging in 25 patients with amnestic mild cognitive impairment (aMCI) and 23 matched healthy controls (HC). While the changes in tract profiles were parallel across aMCI and HC, we found a significant focal shift in the profile at *specific locations* along major tracts sub-serving memory in aMCI. Particularly, our findings depict white matter alterations at specific locations on the right cingulum cingulate, the right cingulum hippocampus and anterior corpus callosum (CC) in aMCI compared to HC. Notably, focal changes in white matter tract properties along the cingulum tract predicted memory and cognitive functioning in aMCI. The results suggest that white matter disruptions at specific locations of the cingulum bundle may be a hallmark for the early prediction of Alzheimer’s disease and a predictor of cognitive decline in aMCI.

## Introduction

Mild Cognitive Impairment (MCI) is a condition characterized by cognitive impairment while retaining intact functioning in everyday life^1^. Individuals with the amnestic subtype of MCI (aMCI), are classified as having progressive memory deficits, which are either isolated or associated with other cognitive domain impairments. Individuals with aMCI are at increased risk for developing Alzheimer’s disease and in some cases, may be in the prodromal stage of Alzheimer’s disease^2^. There is increasing evidence showing that the estimated prevalence of aMCI in population-based studies ranges from 8.4 to 25.2% for individuals 65–85 years old^3^. Despite intense efforts to develop effective medications and pharmacological interventions, there is currently no cure for Alzheimer’s disease (AD). A non-invasive, objective biomarker capable of predicting AD before the onset of more severe cognitive symptoms is therefore crucial for implementing early AD interventions.

Previous research has shown that changes in white matter tissue properties are highly relevant to cognitive decline associated with aging. Apolipoprotein E4 (*APOE4*), the most prevalent genetic risk factor for AD, codes for a protein involved in white matter myelination^4^. Individuals with aMCI typically exhibit more white matter tissue changes related to demyelination compared to controls^5,6^. aMCI patients also show decreased fractional anisotropy (FA) and increased mean diffusivity (MD), which define the directional coherence and the magnitude of water molecule diffusion in white matter tracts, relative to controls^7^. Cortical disconnections as a result of axonal demyelination may explain cognitive decline in aMCI and could be considered a reflection of intrinsic impairments that may lead to AD. When quantified, these white matter abnormalities could plausibly be used for the early identification of individuals at higher risk of developing AD and to plan for early intervention before substantial neurological compromise.

Most recent quantitative investigations of white matter microstructural changes in older adults have focused on diffusion-weighted magnetic resonance imaging (dMRI). dMRI is a non-invasive technique that measures the natural displacement of water molecules (also known as Brownian motion) in brain white matter tissue, allowing white matter structure to be probed and imaged on microscopic scales, providing clues to the changes associated with neurodegenerative states. Results from some recent dMRI studies have identified widespread alterations in distinct white matter fiber tracts in aMCI. Notably, major fiber tracts such as the corpus callosum (CC), inferior fronto-occipital fasciculus (IFOF), and posterior cingulum bundle have shown disruptions in white matter integrity in aMCI patients as well as in AD^5,8,9^. These white matter tracts interconnect frontal, parietal and medial temporal regions and are thought to carry signals that contribute to executive control and memory processes. Thus, the widespread degeneration of white matter may contribute to disruption of efficient communication between functional cortical regions formed through axonal bundles in aMCI patients^10,11^.

Furthermore, several recent neuroimaging studies have shown atypical neural substrates in different components of the memory circuitry in aMCI. They have reported higher degrees of aberrant functional connectivity patterns in aMCI in the posterior cingulate cortex (PCC) in the absence of grey matter atrophy^12^, white matter degeneration in the tracts that sub-serve a part of the connection between the hippocampus and PCC^5^, as well as fiber density reduction in aMCI patients in the posterior white matter cingulum bundle^9^.

For the last decade, there has also been a profound interest in linking individual differences in cognitive test performance to variations in neurobiological substrates in aMCI patients. The majority of these studies have examined regional brain volume properties, brain function, as well as voxel-level white matter properties as predictors of cognitive dysfunction in aMCI^13–16^. More recently, a number of studies have made essential contributions endorsing differences in cognitive performance, to a significant extent, manifested in the properties related to myelin integrity of white matter tissue parameters^17^. Specifically, white matter tract metrics revealed a correlation with semantic verbal fluency, a cognitive function known to decline in AD^18^. Further, structural networks constructed using whole brain tractography exhibited reduced efficiency that was associated with cognitive performance^19^. The studies cited above strongly suggest that measurable changes in white matter accompany behavioral decline in cognition.

*Despite these previous findings, the characterization of how white matter microstructure varies at specific locations along the fiber tracts in aMCI patients remains unclear. This in part reflects the fact that the previous techniques usually extracted a global mean value for each tract and did not capture more fine-grained changes in white matter properties along the tract. In a variety of clinical conditions, when there are group differences in the mean values for a tract*^20^*, it is possible that the difference reflected throughout the entire tract is not sufficiently sensitive to classify an individual’s clinical outcome. Recently, promising progress has been made on diffusion tractography methods to reliably measure fine-grained changes in white matter properties along white matter fibers (tract profile), rather than solely extracting a global mean value for each tract*^21^*. White matter consists mostly of glial cells and myelinated axons carrying signals between various brain regions. Along the major white matter tracts, numerous sets of axons enter and exit at different spatial locations that are relevant for a number of cognitive functions. Thus, summarizing the entire tract with a single diffusion parameter may lead to a loss of valuable information. Using a novel technique, named Automatic Fiber Quantification (AFQ)*^21^*, previous studies have demonstrated that diffusion properties vary significantly along tracts that would capitalize on the precision of tractography for localizing the white matter fiber tracts at different locations in individual patients. Further, it has been shown that the profile of changes in white matter properties along a tract is associated with age during development and these age-related changes in white matter properties occur at specific locations within each tract*^22^. *Lastly, the utility of analyzing white matter properties along tract trajectories has been elucidated in multiple clinical conditions*^23–25^*. Here, we examined if white matter tract profiles in aMCI differed from those of HC and if any observed differences were associated with the memory deficits that hallmark aMCI as an effort toward the detailed characterization of neurodegeneration of white matter tract structure*.

To do this, we conducted a fine-grained examination of white matter tracts for the accurate characterization of how and where white matter microstructures vary in aMCI patients. We first examined alterations in white matter properties along five tracts chosen a priori; the left and right cingulum cingulate, the left and right cingulum hippocampus and anterior CC, all of which are considered part of the core memory circuitry in aMCI. Tract profile approach was used to measure white matter microstructure across the entirety of each tract, enabling localization of specific abnormalities to focal portions of the tracts. This approach allowed us to examine the extent to which any focal abnormalities along each of these white matter tracts are associated with changes in cognitive-behavioral outcomes in aMCI patients.

## Results

### Demographic information and cognitive outcomes

Descriptive statistics of demographic characteristics and neuropsychological assessments are summarized for aMCI patients and in HC in Table 1. aMCI and HC participants did not differ significantly in age, sex, years of education, intracranial brain volume (ICV) or white matter hyperintensity volume (WMHV). aMCI patients demonstrated significantly lower scores in LM-II, MMSE, and SDMT assessments compared to HC, but LSWM and RAVLT scores did not differ significantly between groups. *Compared with normative scores, both HC and aMCI groups scored higher than expected that could be partly explained by the nature of the NIH Toolbox RAVLT assessment as well as very high education level of our sample, among others. Further, our aMCI sample had a marginally higher education level compared with HC that could explain the lack of significant group differences in RAVLT performance in our study. This is supported by previous reports that suggest a significant association between education level and RAVLT performance in older populations*^26^.

**Table 1:** Demographic, clinical and neuropsychological characteristics of the sample.

	HC (N = 23)	aMCI (N = 25)	Statistics
Age, years	72.3 ± 6.2	73.4 ± 6.28	*p* = 0.57
Gender (F/M)	16/7	15/10	*p* = *0.7*
Years of Education (SD)	16.5 ± 1.6	17.6 ± 1.9	*p* = 0.064
Logical Memory-II (SD)	12.4 ± 2.8	6.8 ± 2.3	*p* < *0.001*
MMSE (SD)	29.7 ± 0.5	28.2 ± 1.4	*p* < *0.001*
ICV (SD)	1403 ± 122	1423.6 ± 119	*p* = *0.77*
SDMT (SD)	76.59 ± 15.8	62.4 ± 14.5	*p* < *0.001*
LSWM (SD)	110.7 ± 11.6	105.3 ± 16	*p* = *0.298*
RAVLT (SD)	22.8 ± 5.4	20.9 ± 4.6	*p* = *0.19*
CDR (SD)	0 ± 0	0.26 ± 0.25	*p* < *0.001*
WMHV (SD)	4.85 ± 6	4.01 ± 4.1	*p* = *0.4*

* p values derived from two-sample t-test, Chi-square test or ANOVA with age, gender, education and intracranial volume as covariates.

HC: Healthy Control; aMCI: amnestic Mild Cognitive Impairment; MMSE: Mini-Mental State Examination; ICV: intracranial brain volume; SDMT: Symbol Digit Modalities Test; LSWM: List Sorting Working Memory; RAVLT: Rey’s Auditory Verbal Learning Test; CDR: Clinical Dementia Rating; WMHV: White matter hyperintensity volume (ml).

### Tract-specific group differences

**Figure 1:**
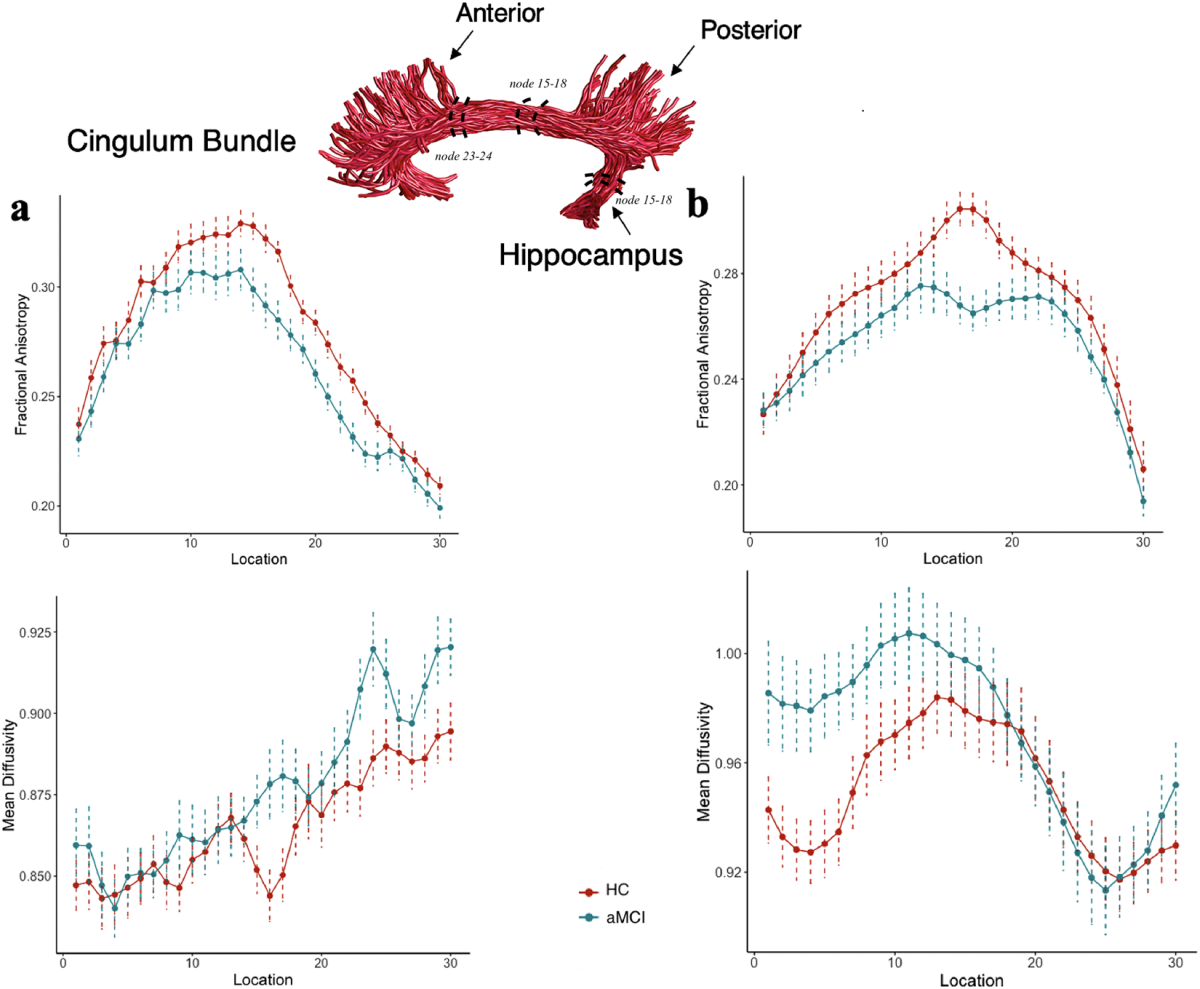
Tract profiles between the 1^st^ and 30^th^ nodes (posterior to anterior) in aMCI and HC for the right cingulum cingulate (**a**) and the right cingulum hippocampus (**b**). Solid lines represent the group average FA and MD across subjects, and dotted lines denote standard error of the mean. Tract rendering is shown for an example subject. The middle 66% (nodes 7–24) of each tract was included in the statistical analysis to avoid partial volume effect that occurs at the end points of the tract. The right cingulum cingulate (**a**) along the nodes 15–18 and 23–24 and the right cingulum hippocampus (**b**) along the nodes 15–18 showed a decrease in FA in aMCI compared to HC (FDR-corrected, p < 0.05). MD was significantly different between groups along the nodes 16–17 and 23–24 only in the right cingulum cingulate (FDR-corrected, p < 0.05).

**Figure 2:**
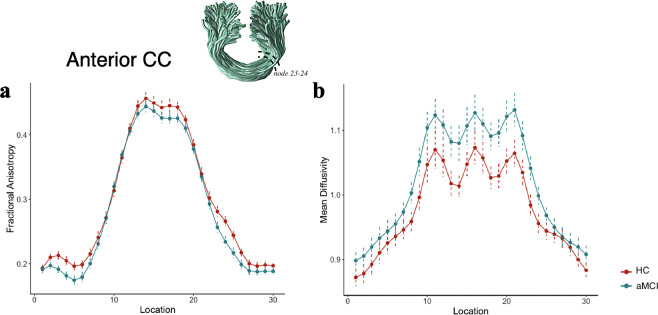
Tract profiles between the 1^st^ and 30^th^ nodes in aMCI and HC for the anterior CC (**a,b**). Solid lines represent group averages for FA and MD across subjects, and dotted lines denote standard error of the mean. Tract rendering is shown for an example subject. The middle 66% (nodes 7–24) of each tract was included in the statistical analysis to avoid partial volume effect that occurs at the end points of the tract. The anterior CC showed a decrease in FA along the nodes 23–24 and an increase in MD along the nodes 18–20 and 23–24 in aMCI compared to HC (FDR-corrected, p < 0.05).

Differences in changes in FA and MD values (commonly derived white matter properties) at middle nodes along the left and right cingulum cingulate, the left and right cingulum hippocampus and corpus callosum forceps minor (the anterior CC) were derived for aMCI and HC. No significant group differences were found for the mean tract values generated from the nodes 7–24. However, significant group differences were observed at the level of single nodes (FDR-corrected, p < 0.05). In the right cingulum cingulate (Fig. 1a), we found significantly lower FA values at nodes 15–18 (posterior and middle) (effect size d = 0.95) and 23–24 (anterior) (effect size d = 0.9) and higher MD at the nodes 16–17 and 23–24 (effect size d = −0.82) in the aMCI group compared with controls (FDR-corrected, p < 0.05). Similarly, significant lower FA at the nodes 15–18 (effect size d = 1.15) in the right cingulum hippocampus (Fig. 1b) were present in the aMCI group compared to HC (FDR-corrected, p < 0.05). MD values along the right cingulum hippocampus were not significantly different between groups (FDR-corrected, p < 0.05). For the left cingulum cingulate, significantly higher MD at the nodes 19,20 and 23 were observed in aMCI patients compared to HC. However, this significance did not survive FDR correction. For the left cingulum hippocampus, no significant difference was found between aMCI patients and HC at any of the nodes. For the anterior CC, the aMCI group displayed significantly lower FA at nodes 23–24 (right anterior CC) (effect size d = 0.85) and higher MD at nodes 18–20 and 23–24 (effect size d = −0.78) compared to HC (see Fig. 2).

*In a subsequent exploratory analysis (refer* to supplementary material*), we also examined white matter group differences in several additional tracts previously shown to be disrupted in aMCI patients, including in the posterior corpus callosum (posterior CC), IFOF, and corticospinal tracts*^27^. As shown in Supplementary Figure 1, the right IFOF showed a decrease in FA along the nodes 17–19, and the posterior CC showed an increase in MD along nodes 9–10, 14–16, and 20 in aMCI compared to HC (p < 0.05, uncorrected). Decreased FA at node 18 of the right IFOF tract in aMCI remained significant after FDR correction (p < 0.05, FDR-corrected). The left IFOF displayed a lower FA value along nodes 16–19 in aMCI patients (p < 0.05, uncorrected). No significant group difference was observed in MD for any nodes along the bilateral IFOF or in FA along the posterior CC. Moreover, no significant group difference was observed in FA or MD at any node along bilateral corticospinal tracts (p < 0.05, uncorrected).

We also observed that MD was the most age-sensitive parameter. Positive age associations with MD were observed along the nodes presenting significant group differences between aMCI and HC in the right cingulum cingulate, anterior CC and posterior CC, (p < 0.05, FDR-corrected)  which is in line with the previous study^28^.

### Association between tract properties and cognition 

**Figure 3:**
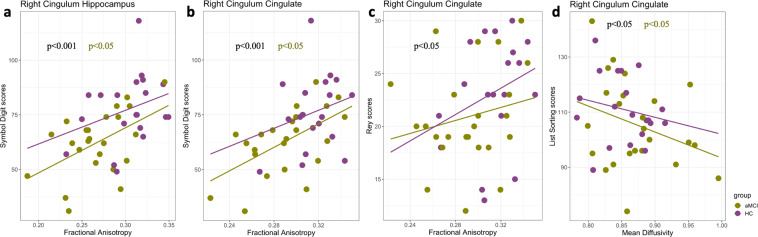
Correlation analysis quantifying brain-behavior associations at the specific tract level. Scatterplots illustrating the relationship between FA (mean values derived from the nodes significantly different between groups) and SDMT scores in the right cingulum hippocampus (**a**) and the right cingulum cingulate (**b**), the relationship between mean FA and RAVLT scores in the right cinculum cingulate  (**c**), and the relationship between MD (mean values derived from the nodes significantly different between group) and LSWM scores in the right cingulum cingulate (**d**). Results are based on correlation analysis with age, sex and ICV as controlling variables. Statistically significant correlations (p < 0.05, FDR-corrected) are presented with black color for the full dataset. Statistically significant within group correlations (p < 0.05, FDR-corrected) are presented in purple (HC group) and green (aMCI group).

We first examined the association between the properties of white matter tracts that are crucial components of memory circuitry and cognitive functioning. Multiple regression analysis revealed that SDMT scores were positively correlated with mean FA value over the nodes 15–18 (t = 3.75, p < 0.05, FDR-corrected) and nodes 23–24 (t = 2.78, p < 0.05, FDR-corrected) in the right cingulum cingulate and with the mean FA value over nodes 15–18 in the right cingulum hippocampus (t = 4.46, p < 0.05, FDR-corrected) across groups (Fig. 3a,b). However, SDMT scores were not significantly correlated with MD values at any nodes in the right cingulum cingulate. Within the aMCI group, mean FA for nodes 15–18 and 23–24 in the right cingulum cingulate and nodes 15–18 in the right cingulum hippocampus were significantly correlated with SDMT scores (p < 0.05, FDR-corrected). These associations were not significant within the control group or between groups. Further, in the right cingulum cingulate, mean MD values for nodes 16–17 were negatively correlated (t = −2.387, p < 0.05, corrected) with LSWM scores across groups as well as within the aMCI group (Fig. 3d). Similar effects were observed when examining correlations between LSWM scores and mean MD derived from the nodes 23–24 in the anterior CC. In contrast, we found no significant association between LSWM scores and mean MD values derived from the nodes 23–24 in the right cingulum cingulate or with nodes 23–24 in the anterior CC. Finally, mean FA (nodes 15–18) in the right cingulum cingulate showed a significant positive relationship with RAVLT scores across groups as shown in Fig. 3c ((t = 2.24, p < 0.05, FDR-corrected). The reported associations were also observed at each node that showed significant group differences (p < 0.05, FDR-corrected).

In a posteriori analysis, we also examined the associations between additional white matter tract parameters and cognitive test scores. However, we did not find any significant associations between white matter properties (at single node level or average) and cognitive test scores for these tracts.

## Discussion

Amnestic Mild Cognitive Impairment (aMCI) was associated with alterations in tissue properties at specific locations along three white matter tracts considered crucial for memory: the right cingulum cingulate, right cingulum hippocampus and anterior CC. Particularly, we found focal white matter changes in aMCI throughout the cingulum tract that has multiple cortical associations. Further, these focal changes in white matter tissue properties along the cingulum tract were linked to cognitive performance with a varying magnitude in aMCI patients. Notably, higher SDMT scores were associated with increased myelination in specific parts of the right cingulum cingulate (along the nodes 15–18 and 23–24) and right cingulum hippocampus (along the nodes 15–18) in aMCI patients. Similarly, lower LSWM scores were associated with demyelination along the nodes 16 and 17 in the right cingulum cingulate in aMCI. Finally, RAVLT scores were also positively correlated with mean FA values (along the nodes 15–18) in the right cingulum cingulate across groups. The results suggest that early white matter alterations in specific parts of the cingulum tract may be predictive of preliminary stages of cognitive decline in AD. This study adds to our understanding of the white matter microstructure of the brain with more precise spatial localization that provides higher sensitivity for detecting abnormalities in aMCI patients.

Our results indicated that white matter integrity was disrupted in the right cingulum cingulate and right cingulum hippocampus at multiple locations along these tracts in aMCI patients compared to HC(Fig. 1a,b). dMRI studies have consistently identified involvement of the cingulum white matter tract in aMCI and AD. The observed network shows good topographic overlap with areas affected in aMCI, specifically, in the posterior cingulum cingulate. Significantly reduced FA, increased MD and reduced fiber density have been previously reported in the posterior cingulum cingulate in aMCI^5,9,29^. While these observations are in line with our findings, neither of these studies reported white matter abnormalities in the anterior cingulum cingulate. The cingulum bundle is a prominent white matter tract that incorporates various brain regions including those found within the default mode network (DMN),  as well as with temporal and parietal brain regions. Its degeneration may thus contribute to the impairment of several cognitive functions. We speculate that the previous methods applied to examine white matter integrity across the entirety of the tract might have precluded identification of any focal abnormality in the cingulum cingulate. Using the tract profile approach, our data further demonstrate that the right cingulum hippocampus shows significantly lower focal FA values in the middle section of this tract in aMCI (see Fig. 1b). Previous studies have reported alterations in the hippocampal cingulum fiber only after the onset of AD symptoms^9,30^. Our findings thus provide a unique window into white matter alterations in aMCI that precede clinical symptoms of AD.

*One recent study using the same method observed that only the middle part of the right cingulum cingulate and left arcuate fasciculus were affected (lower FA and higher MD) in MCI patients*^31^*. However, we have found several locations along the right cingulum cingulate, as well as along other tracts, that were affected in aMCI patients. It should be noted that the diffusion sequence used in our study has a higher number of directions (119) with multiple b values compared to the sequence used in Zhang et al. (2019) study (32 directions with a single b value). Further, we have applied a DK tensor model that accounts for the non-Gaussian behavior of water molecules in heterogeneous tissue and Fiber Orientation Distribution (FOD) that provides an estimation of the orientation distribution of the fibers in each voxel to generate whole brain tractography using the Anatomically Constrained Tractography (ACT) probabilistic algorithm. Overall, our data suggest widespread changes in white matter tract properties in aMCI and provide further insights regarding the relationship between the observed white matter changes and behavioral outcomes*.

Anterior and posterior CC and IFOF fibers interconnect the anterior and posterior components of DMN regions^32^. Previous evidence indicates that functional DMN network abnormalities track AD progression and are associated with disease severity^33–36^. However, white matter damage in aMCI is not confined to the DMN, but typically extends to frontal regions as well^8,37,38^. Our results revealed significantly reduced FA in the right anterior CC (Fig. 2) (which projects into the medial and lateral frontal cortices) and the right IFOF (Supplementary Fig. 1a). Our results also revealed increased MD in the right anterior CC (Fig. 2b) as well as in the posterior CC (which projects into the posterior parietal and occipital cortices) (Supplementary Fig. 1b). These findings are consistent with other studies on preclinical AD reporting decreased fiber tract integrity within the anterior CC, posterior CC, and IFOF tracts several years before the presumed onset of AD symptoms. Our findings further support the possibility that callosal degeneration in white matter integrity contributes to the disruption of functional networks such as the DMN^39,40^ even though any correlational data preclude a causative conclusion.

*These observations highlight that there is a substantial white matter degeneration in the right part of the brain in aMCI patients. To date, there has been plenty of neuroimaging research documenting the atrophy and white matter changes in the right hippocampus and cingulum bundle in aMCI and AD participants*^41–43^*. Decreased right hippocampal volume was associated with poorer cognitive performance and increased tissue loss (lower FA, higher MD, and lower fiber density) in the right cingulum bundle, all of which have been consistently reported in aMCI and AD.*^41,44^*. Thus, our results support that the right hippocampus and cingulum bundle play a critical role in cognition, particularly when cognitive impairment is present, and the results can serve as a basis for future research to ascertain the ability to identify patients in the preclinical stage of AD*.

Extending the growing trend for dMRI studies, the measurements in this study offer a different view on the white matter that enables us to quantify tissue integrity and its relationship with cognitive skills at multiple locations along each fiber. Since cognitive skills depend on the communication of distributed cortical networks, cognitive decline may have widespread consequences across the white matter tracts. Most of the functional changes that have been observed in aMCI were prevalent and associated with cognitive decline or disease progression. It has been suggested that synaptic connectivity and functional brain networks are strongly linked and share many organizational principles in favor of efficient information exchange between cortical regions^11^. However, some evidence suggests that structural connectivity remains stable while functional connectivity is remodeled. Thus, relatively small and focal structural modifications could result in widespread functional changes, although a consensus has not yet been established regarding the relationship between structural and functional network changes in neurodegenerative diseases.

Perturbation of myelin growth and pruning have grave consequences on cognitive performance in aMCI patients. For example, reduced myelin integrity of white matter results in damages to the myelin sheath, which is associated with pathologies characterized by low cognitive skills in aMCI and AD^17,44–46^. These observations are in line with the results obtained from our experimental data. Within the aMCI group, abnormalities in these white matter tracts were linked to changes in cognitive performance (Fig. 3). SDMT scores, central to several cognitive skills and particularly requiring rapid communication between cortical regions, were highly correlated with FA values in multiple locations along the right cingulum cingulate and the right cingulum hippocampus. Further, LSWM scores were negatively correlated with MD values in the right anterior CC and in the right middle cingulum cingulate. These findings are consistent with the hypothesis that individuals with higher FA or lower MD (better myelination) are more likely to display higher cognitive abilities in aMCI patients. It should be noted that of the tract abnormalities identified in aMCI, the anterior CC, right cingulum cingulate and right cingulum hippocampus carry signals not only related to memory but also relevant to several other cognitive functions. Given the relatively coarse (mm) scale of white matter, it is possible to investigate how cognitive decline affects distinct sub-populations of fibers with discrete cortical terminations and functional nodes.

One concern of the present study is the relatively small sample size, and thus the generalizability of the results is somewhat limited. Future longitudinal studies on a more substantial number of aMCI patients will allow us to corroborate our findings. Another potential extension of our work would be to integrate multiple imaging modalities to address the possible interplay between structural and functional network changes. These additions will allow us to follow patients with aMCI longitudinally to determine progression to AD. Next, the classification of participants into aMCI and control groups was solely based on neuropsychological measures and lacked biological markers of AD such as measurements of beta amyloid and neurofibrillary tau deposition. Nevertheless, all patients underwent extensive aMCI screening using clinical and neuropsychological examinations. Finally, because of limited resources, we only had Clinical Dementia Rating (CDR) information on a subset of participants. We tested the differences in cognitive profiles (e.g. MMSE, LM-II, SDMT, LSWM and RAVLT scores) and mean FA values that showed significant between-group differences between aMCI participants with (*N* = 11) and without (*N* = 14) CDR scores and found no significant differences between them (Supplementary Fig. 2).

In summary, the results of the present study provide a unique contribution to our understanding of white matter integrity and its association with behavioral performance in aMCI patients. Particularly, we observed changes in both FA and MD along the cingulum tract in aMCI compared to HC, suggesting the disorder starts with an initial failure of multiple regions along the cingulum bundle connecting distinct parts of the memory circuitry. The observed pattern of disruptions of the white matter tract profile may shed light on how Alzheimer’s disease progresses and affects information transfer among brain regions.

## Methods

### Participants

Twenty-five patients with aMCI (age range 65–85 years) and 23 age- and education-matched healthy controls (HC) (age range 65–85 years) were included in the analysis for this study (Table 1). At the time of initial recruitment, all participants were screened for current and past history of medical and psychiatric conditions through the use of an electronic screening form. Exclusion criteria for all participants included; left-handedness, presence of suicidality, formal diagnosis of a significant psychiatric disease, current regular use of psychotropic medications, opiates, or thyroid medications (except for permitted medications including cholinesterase inhibitors and hypertension medications if stable for at least two months), claustrophobia, non-MRI-compatible materials, current substance abuse, post-traumatic or psychotic disorders, bipolar disorder; any significant neurologic disease, including possible and probable dementia, multi-infarct dementia, Parkinson’s or Huntington’s disease, brain tumor, progressive supranuclear palsy, seizure disorder, subdural hematoma, multiple sclerosis, “uncontrolled” hypertension, history of significant head trauma, history of alcohol or substance abuse or dependence within the past 2 years; any significant systemic or unstable medical condition.

Following consents, eligible participants then completed a battery of neuropsychological assessments before study enrollment to further determine study eligibility. Cut-off scores on the M.I.N.I (mini international neuropsychiatric interview)^47^, a structured clinical interview screening for primary psychiatric conditions, the geriatric depression scale (GDS), used as a depression screener, the Mini-Mental State Examination (MMSE), indicative of global cognition, and the instrumental activities of daily living (IADL), assessing functional ability on eight independent activities of daily living, were used to determine study eligibility. Inclusion criteria for both groups included MMSE scores >=24, GDS < = 7 and an intact IADL. The Logical Memory II-Delayed Recall (LM-II) subset of the Wechsler Memory Scale for episodic memory was used to categorize participants as either having aMCI (LM-II subscale cutoff scores of <=8, <=4 and <=2 for 16, 8–15 and 0–7 years of education) or HC (LM-II subscale cutoff scores of >=9, >=5 and >=3 for 16, 8–15 and 0–7 years of education). The Stanford University Institutional Review Board approved all the data collection procedures, and all experiments were performed in accordance with relevant guidelines and regulations. The informed consent was obtained from legally authorized representative for only amnestic mild cognitive impairments (aMCI) patients and informed consent was obtained from healthy participants.

### Cognitive measures

The National Institutes of Health (NIH) Toolbox Cognition domain (nihtoolbox.org) is a computerized iPad-based toolbox for assessing cognitive functions along multiple domains. The Symbol Digit Modalities Test (SDMT), List Sorting Working Memory (LSWM) Test, and Rey's Auditory Verbal Learning Test (RAVLT) were among the assessments administered for this study. SDMT measures processing speed, which is central to many cognitive functions. The SDMT consists of a series of nine symbols presented at the top of a standard sheet of paper, each paired with a single digit. The rest of the sheet contains symbols not matched to a digit, and participants were asked to say the digit corresponding with each symbol as quickly as possible and without making mistakes. The resulting score is equal to the number of correct matches over a period of 120 seconds. LSWM is a list sequencing test assessing working memory that requires participants to sort and sequence auditorily and visually presented stimuli. The NIH Toolbox version of RAVLT is a computerized adaptation of RAVLT (www.parinc.com), a measure of immediate recall for unrelated words presented via audio recording, where the participant is asked to recall as many words as possible following the auditory presentation of the same list of words over three trials.

### MRI acquisition and processing

All diffusion tensor imaging (DTI) data were collected on a 3 T GE system (General Electric Healthcare, Milwaukee, WI, USA) equipped with a 32-channel head coil (Nova Medical, Wilmington, MA, USA) using a multiband echo-planar imaging (EPI) acquisition scheme (multiband factor of 3, MB = 3) at the Center for Cognitive and Neurobiological Imaging at Stanford University (http://www.cni.stanford.edu/). Multi-shell diffusion data were acquired for all participants, with isotropic 2.0 mm^3^ spatial resolution in 80 diffusion directions, and diffusion gradient strength was set to b = 2855 s/mm^2^ and 30 diffusion directions with diffusion gradient strength b = 710 s/mm^2^ ^48^. Each DTI image also contained nine images without diffusion weighting (b = 0 s/mm^2^). An additional scan was acquired in the opposite phase encoding direction, consisting of 6 diffusion directions (b = 2855 s/mm^2^) and yielding two non-diffusion-weighted images for EPI distortion correction. Other DTI parameters are as follows: TR/TE = 2800/78 ms, matrix size=112 × 112, and 63 axial slices. T1-weighted images were acquired in the sagittal plane using a three-dimensional GE’s BRAVO sequence at a resolution of 1 × 1 × 1 mm with a 256 × 256 field of view. ICV for all participants was calculated on T1-weighted images using FreeSurfer Software Suite, version 6.0 (http://surfer.nmr.mgh.harvard.edu/). DTI data preprocessing was implemented in FSL (fsl.fmrib.ox.ac.uk/fsl/fslwiki/) and MRTrix3(mrtrix.org) and included denoising, geometric EPI distortion correction (FSL’s topup function), eddy current distortion correction, slice-by-slice motion correction, outlier detection and bias field correction (ANTs N4BiasField Correction). After preprocessing, a tensor model was fitted to  each voxel’s data using the diffusion kurtosis (DK) model, with the adjusted diffusion gradients accounting for the rotation applied to the measurements during motion correction. DK is an extension of the diffusion tensor model that accounts for the non-Gaussian behavior of water in heterogeneous tissue containing multiple barriers to diffusion that may be useful for investigating neurodegenerative diseases^49^. The fiber orientation distributions (FODs) were generated on an aligned and distortion corrected DTI data using multi-shell, and multi-tissue (white matter, grey matter, and CSF) constrain spherical deconvolution using the average tissue response function in MRtrix. To quantify the potential effects of motion, data were manually checked for imaging artifacts, and participants with mean slice-by-slice root mean square displacement values of >1.2 mm were excluded from the analysis. No significant difference was observed in motion between aMCI and HC (p = 0.084). Further, the motion was included as a covariate, which did not change the results.

White matter hyperintensities (WMH) can be detected using T2-FLAIR images of the brain in older people. The underlying WMH pathology reflects demyelination and axonal loss^50^. To evaluate and localize WMH, T2-FLAIR images were acquired using a single-slab 3D fast spin-echo (Cube FLAIR) sequence with inversion recovery pulses at inversion time (TI)/echo time (TE)/repetition time (TR) = 1878/120/6500 ms, field of view FOV = 256 × 256, with a slice thickness of 1 mm. The presence of the lesions around the cingulum bundle affected the tract segmentation in HC and aMCI patients. The data from one HC and two aMCI patients were removed due to the lousy segmentation in the cingulum cingulate tract.

### White matter tract identification

FA and MD are the most widely used white matter metrics in neurodegenerative disorders. These metrics describe the directional coherence and magnitude of water diffusion, respectively. Water molecules tend to diffuse with greater directional coherence in the presence of well-myelinated axons, and FA is increased. Tissue loss (demyelination) lowers the local barriers for the movement of water molecules, causing an increase in the magnitude of diffusion, and MD is increased. Thus, the abnormal FA and MD observed in aMCI along brain white matter tracts reflect meaningful differences in underlying microstructure.

The Automated Fiber Quantification (AFQ)(v1.1) (github.com/yeatmanlab/AFQ/wiki) software package was used to segment fiber tracts and quantify tract profiles of tissue properties along each tract^21^, after initial generation of a whole-brain connectome using probabilistic tractography in MRtrix (3.0) using white matter FOD image of each subject. The AFQ uses a two way-point region of interest (ROI) procedure to define 20 white matter tracts, in which a fiber from the whole-brain connectome becomes a candidate for a specific fiber group if it passes through two ROIs representing the trajectory of the fiber group^51^. Each candidate fiber passing through regions of white matter unlikely to be part of the tract were removed to define all the tract fibers at the central position^52^. Fibers more than three standard deviations from the mean fiber length were removed. The diffusion metrics were then projected onto the segmented white matter fibers generated by AFQ. Thirty evenly spaced nodes along each fiber were created on selected tracts, spanning termination at the grey-white matter boundary. Finally, rather than computing the mean diffusion properties (MD and FA) for each tract, diffusion properties were calculated using a weighted sum of each fiber’s value at a given node based on its Mahalanobis distance from the core location of the tract^21^. Tract profiles can be averaged to produce a single mean value for each tract, or models can be fitted at each node along the tract profile. This improves detection power when the pathology is more pronounced in a specific part of the tract in patients compared with controls^53^. It is noteworthy that the extracted tract profiles in the present study were consistent with those reported in previous studies in typical and atypical individuals^21,52,54^. This consistency demonstrates the precision of the method applied in this study for quantifying tissue properties at multiple equidistant nodes along a fiber tract.

Initially, five tracts were investigated using AFQ. Investigated tracts included the left and right cingulum hippocampus, the left and right cingulum cingulate and the anterior CC, all of which are thought to connect the core memory and cognitive circuitry of the brain^55,56^. *The fornix is also a memory-related tract*^55,57^ *that is shown to be affected in AD and aMCI*^58,59^*. We used the JHU-white matter atlas*^51^ *to segment whole brain tractography into 20 different white matter tracts. Unfortunately, the JHU atlas does not include fornix tract. F*or exploratory analysis, we included five additional tracts previously shown to be disrupted in aMCI patients: the right and left IFOF, the right and left corticospinal tracts and the posterior CC, albeit less consistent across studies.

### Statistical analysis

All statistical analyses were carried out using software written in R (version 3.5.3). To eliminate the influence of crossing fibers near cortical terminations and to avoid partial volume effects at grey-white matter boundaries, diffusion properties were first averaged over the middle sections (nodes 7–24) of each tract to generate a single mean diffusion parameter for each tract. Analysis of covariance was then applied to the resulting mean values from the previous step to test the main effect of group (aMCI vs. HC), with age, sex, ICV and years of education as covariates. The same approach was used to analyze between-group differences on the level of single nodes. FDR was carried out across 18 nodes across tracts. To further examine the relationship between fine-grained tract properties and behavior, a multiple regression analysis was performed using SDMT, LSWM, and RAVLT scores as dependent variables and mean tract values, age, sex, age-sex interaction and education level as independent variables. Notably, the single white matter properties of each node and the mean tract values derived from the portion of the tracts showing significant group differences were entered into the multiple regression analysis. For all reports, FDR correction was applied to the p values to correct for multiple comparisons (corrected alpha = 0.05).

## Supplementary information

Supplementary Information.
