# Supplementary material for: Focal white matter disruptions along the cingulum tract explain cognitive decline in amnestic mild cognitive impairment (aMCI)
Source: Sci Rep. 2020 Jun 23;10:10213. doi: 10.1038/s41598-020-66796-y (PMC7311416; doi:10.1038/s41598-020-66796-y)
Supplement: Supplementary file 1 — Supplementary Information. [file 41598_2020_66796_MOESM1_ESM.docx]

**Supplementary material for “Focal white matter disruptions along the cingulum tract explain cognitive decline in amnestic mild cognitive impairment (aMCI)”**

Elveda Gozdas^1^, Hannah Fingerhut^1^, Lindsay Chromik^1^, Ruth O’Hara^1^, Allan L. Reiss^1^, S.M. Hadi Hosseini^1^

**^1^**Department of Psychiatry and Behavioral Sciences, Stanford University School of Medicine, Stanford, CA

**Supplementary Figure 1.**


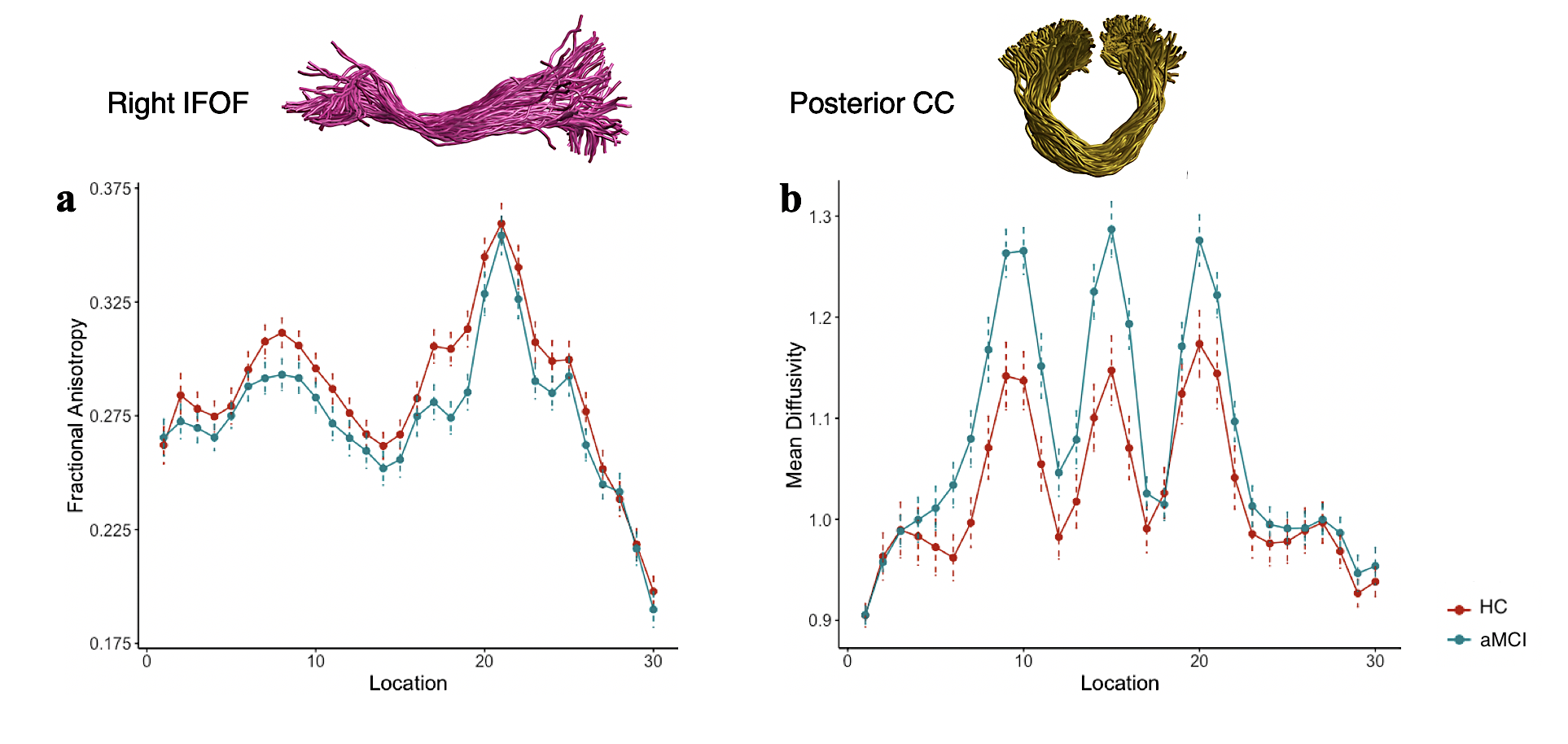


Tract profiles between 1^st^ and 30^th^ nodes in aMCI and healthy controls (HC) for left IFOF (Inferior Fronto-Occipital Fasciculus) and the posterior (forceps major) corpus callosum (CC) (a, b). Solid lines represent the group average fractional anisotropy (FA) and mean diffusivity (MD) across subjects and dotted lines denote standard error of the mean. Tract renderings are shown for an example subject. The middle 66% (nodes 7-24) of each tract was included in the statistical analysis to avoid partial volume effect that occurs at the end points of the tract. The right IFOF showed a decrease in FA along the nodes 17-19 (node 18, FDR-corrected, p<0.05) and posterior CC showed an increase in MD along the nodes 9-10, 14-16 and 20 in aMCI group compared to HC (p<0.05, uncorrected).

**Supplementary Figure 2.**

**
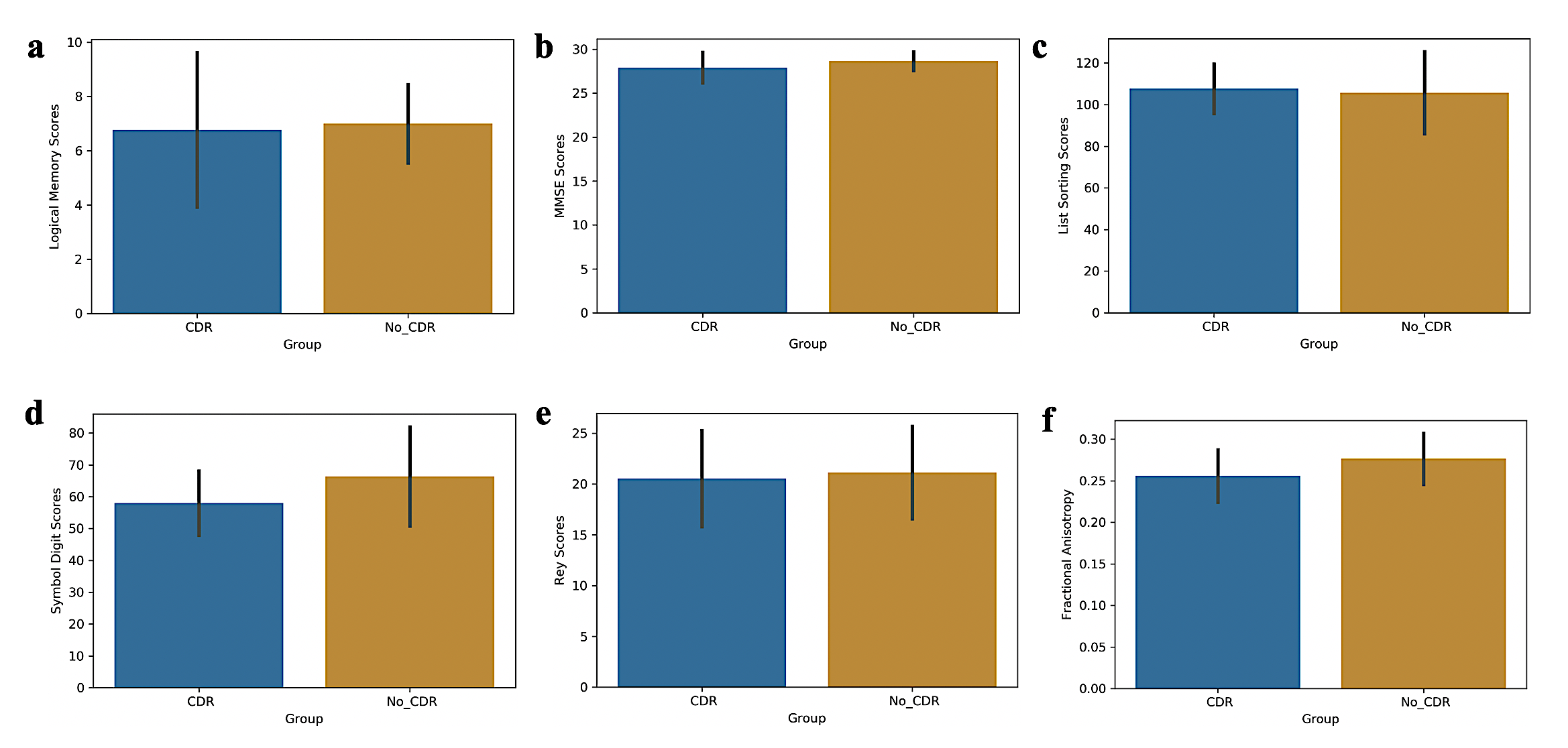
**

Cognitive profile and white matter property did not change between aMCI patients with CDR score 0.5 (*N* = 11) and aMCI patients without CDR (*N*=15) scores. We have tested the differences in cognitive profile (LM-II (a), MMSE (b) and NIH Toolbox test scores (c, d, e)) and mean fractional anisotropy (FA) (nodes 15-18 in the right cingulum hippocampus) (f). Any of the cognitive scores and mean FA did not differ across the groups (p>0.1, uncorrected).
